# Scientific Articulation during Collaborative Digital Game-Based Learning Enhances Learning of Immunology

**DOI:** 10.4049/immunohorizons.2300004

**Published:** 2023-11-01

**Authors:** Pey-Yng Low, Gaik-Bee Lim

**Affiliations:** * School of Life Sciences & Chemical Technology, Ngee Ann Polytechnic, Singapore, Singapore; † Centre for Learning & Teaching Excellence, Ngee Ann Polytechnic, Singapore, Singapore

## Abstract

Digital game-based learning has been used to help learners grasp complex concepts in science subjects such as immunology. The aim of this study was to examine whether playing a digital game collaboratively would encourage articulation of scientific terminology and concepts, and whether this would result in learning gains. Forty-seven students at a tertiary institution (17–19 y of age) played a game (*n* = 22) or watched a video of the game (*n* = 25) in small groups. This was followed by an activity to document the key learning points. Pretest and posttest results showed that although both groups had learning gains, the game-based learning group outperformed the video group for gains in procedural knowledge, suggesting that playing the game helped students to better internalize the steps involved in the immune response. For the game-based learning group, there was a positive correlation between the number of scientific terms articulated and the gains in the test scores. However, for the video group, there was no correlation. The implications for designing digital game-based learning activities for learning are discussed. The study was carried out in an online environment due to the COVID-19 pandemic mandating home-based learning at the time. The discussion also focuses on how the findings can be applied in an online and face-to-face context.

## Introduction

Immunology is a challenging subject to teach and learn. Challenges for first-time learners include having to understand specialized terminology, abstract concepts, and complex processes that are interlinked. Furthermore, the immune responses are dependent on the characteristics and location of the pathogen, and whether it is the first or subsequent exposure. Traditionally, immunology is taught over a period of time, through didactic lectures that follow a sequence of topics as presented in immunology textbooks. However, learners also need to understand how the different topics are connected to appreciate how individual immune components work to orchestrate the immune response during an infection.

Varied approaches have been used to supplement didactic instruction and help learners to grasp basic immunological concepts. These include animations and simulations (1) to enable learners to visualize the immune system components and processes, and active learning approaches to help learners construct knowledge, such as interactive response systems and concept maps (2). One example of an active learning approach is the use of physical objects and manipulatives such as LEGO blocks (3). This introduces a kinesthetic element to the learning experience and has been shown to help learners better understand the immune system components and processes.

Digital game-based learning is another active learning approach that has been used for teaching immunology. The use of digital games for education has been advocated since the early 2000s (4, 5), and studies have shown that digital games can support learning of science (6–9) through the enhancement of conceptual learning and the development of high-level cognitive skills. One advantage of using digital game-based learning for learning immunology is that abstract concepts can be made more concrete through visualizing and manipulating the various immune system processes. Concepts in immunology also lend themselves well to game scenarios where there is a notion of a battle between opposing forces and having to adopt different strategies to counter different “enemies.” This can help students to grasp the big picture of the immune system response as the different components interact together during the immune response to pathogens. Gamification elements can also help engage some students. These include game challenges or objectives that students must achieve to pass a level, a star system for achievements, and leaderboards to foster competition between students.

Several studies have shown that digital games can improve learning of immunology for both younger learners (10, 11) as well as at the undergraduate level (12–15). Although some of these games are bespoke and not publicly available (14), or not in the English language (10), there are several games suitable for tertiary education that are freely available on the Internet, shown in Table I.

**Table I.** Immunology games available on the Internet as of January 2023

Immunology Games	Web Site	Reference	Comments
*Pathogen Attack*	https://gtac.edu.au/students/learning-resources/gtacs-immunology-game/		Gene Technology Access Centre, a specialist science education center in Victoria, Australia
*Immune Attack*	https://melanieanns.itch.io/immune-attack	(9)	
*Immune Defense*	https://melanieanns.itch.io/immune-attack		
*Immune Quest*	http://immunequest.com/	(10)	Only innate immunity components, that is, macrophages, complement, and neutrophils, are available at present

Although digital games can provide a realistic setting for experimentation and situated understanding (5, 16), the key ideas and relationships conveyed through game visuals and mechanics for scientific learning tend to be tacit. This presents a challenge for learners to translate what was learned through gameplay into explicit understanding of disciplinary knowledge (17). Various lines of evidence have suggested that games may not be effective in achieving the intended learning outcomes when used as a standalone activity (18–22). Hence, scaffolding of game-based learning experiences is essential to ensure that learning will extend beyond the boundaries of the game and translate into formal learning of disciplinary content.

Collaborative game-based learning has also been shown to enhance learning of science subjects (23–25). Compared to playing individually, playing games in pairs led to better results in performance-based transfer tasks, demonstrating that collaboration facilitated the transfer of knowledge acquired through gameplay to other situations (23). Peer interactions during collaborative game-based learning could also allow groups to achieve a shared conceptual understanding by providing opportunities for clarification of misconceptions (26, 27), collaborative sense making (23, 26, 28), problem solving (23, 24), and invoking deeper conceptual understanding (29).

More critically for a terminology-heavy subject such as immunology, collaborative learning during science classes may promote the usage of scientific terminologies as learners engage in discussion. The acquisition and articulation of scientific terminology are critical parts of forming the network of concepts that underlie scientific knowledge. A study applying the theory of cognitive linguistics has implicated the role of scientific articulation in physics concept formation, as the test score for concept comprehension was positively correlated to the number of words with scientific meaning in a word association test (30). Another study by Krontiris-Litowitz (31) indicated that a combination of discussion and writing tasks led to improved learning in undergraduate anatomy and physiology.

We had previously developed a single-player digital game for learning immunology, *Mission: Immunity!*. The game was intended for life science diploma students, 17–19 y of age, to help students grasp the big picture of the immune response to a viral and a bacterial infection (14, 32). The game was played in class individually, but there was a social element as students helped each other figure out how to play the game, and friendly competition due to the inclusion of a leaderboard. This was followed by a verbal debrief following gameplay.

In 2020, the COVID-19 pandemic led to the pivoting of in-class tutorials to online lessons. The social element of game-based learning would no longer apply, as students would be playing the game on their computers at home. We took this opportunity to redesign the game-playing experience from an individual to a group activity, to bring in the element of collaborative learning. To encourage collaborative scientific articulation, after the gameplay segment, students engaged in a discussion and recorded their learning points in a written format.

The research questions investigated were as follows: 1) Would the implementation of collaborative game-based learning in a virtual environment enhance the learning of immunology concepts and procedures, compared with watching a video of the game tutorial? 2) Would the articulation of scientific terms correlate with the achievement of specific learning outcomes during a digital game-based learning experience?

## Materials and Methods

### Mission: Immunity! *digital game*

**FIGURE 1.**
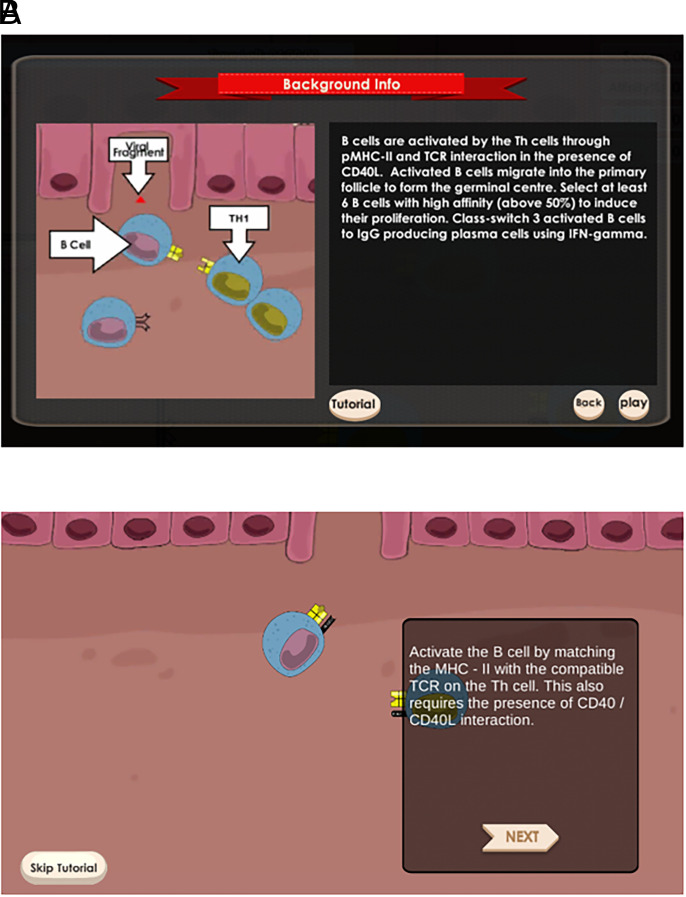
Background information and game tutorial of challenge 8 in the influenza scenario. (**A**) Background information. (**B**) Game tutorial.

*Mission: Immunity!* is a single-player digital game focused on the immune response to infection (14). There are two scenarios, “influenza” and “pneumonia,” broken down into 10 and 6 game challenges, respectively, with each representing a different stage of the immune response. Each game challenge has specific learning outcomes aligned to the curriculum of the immunology module. Before the start of each challenge, students are presented with background information and the game objective of that challenge (Fig. 1A). A game tutorial video without voiceover provides stepwise guidance on how to play the challenge. Content knowledge is embedded in the tutorial (Fig. 1B).

### Participants

The participants were diploma students 17–19 y of age, taking the immunology module in their second year of study at a polytechnic in Singapore. Of the 72 students from six classes, three classes with 39 students were allocated to the experimental condition while another three classes with 33 students were allocated to the control condition based on convenience sampling. The study was approved by the Polytechnic Institutional Review Board (project code NPIRB-P0191-2020-LSCT-LPY26). Parental consent was sought for the students to participate in the study, as they were <21 y of age, according to institutional guidelines. Consent was obtained for 22 students and 25 students from the experimental and control conditions, respectively. Only data from students whose parents had given consent were used. For the collaborative activity that was conducted in groups of three, there were five trios from the experimental condition and six trios from the control condition where consent was obtained for all students. Recordings from these groups were transcribed and analyzed.

### Experimental design

**FIGURE 2.**
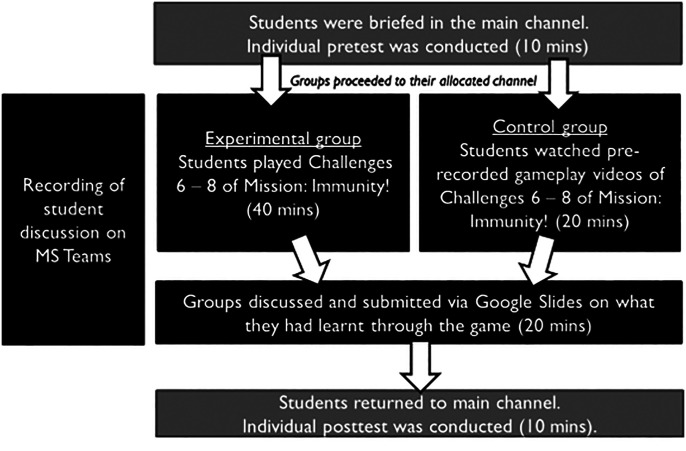
Overview of learning activity for experimental and control groups.

The study was conducted during a regular 2-h online tutorial session. An overview is shown in Fig. 2. Students had been instructed to go through relevant online learning materials on adaptive immune responses prior to the class. During the tutorial, students were briefed regarding the in-class activity and were given 10 min to complete an online pretest survey. Subsequently, students were assigned to breakout trios. For the experimental conditions, students played challenges 6–8 of *Mission: Immunity!* for 40 min. As the game was a single player game, the group member playing the challenges shared his or her screen while the other two members contributed to the discussion on how to pass the challenges. The challenges revolved around the activation of adaptive immune responses that were covered in the self-directed online learning content. The learning outcomes of challenges 6–8 are as shown in Table II. For the control condition, students watched prerecorded gameplay video tutorials (Supplemental Table I), which did not have voiceovers, from the same game challenges played by the experimental groups. Subsequently, both experimental and control groups were given 20 min to discuss and document what they had learned through the gameplay and video, respectively, in an online collaborative document. The discussions and screen activity were recorded. Afterwards, students reconvened in the large group where they were given 10 min to complete a posttest survey. For a given tutorial class session, all students in the class went through either the experimental or control condition.

**Table II.** Learning outcomes of challenges 6–8 of *Mission: Immunity!*

Challenges	Learning Outcomes
6	To describe the Ag presentation pathways involved in the presentation of viral Ags on MHC-I and MHC-II
7	To describe the signals for CD8 or CD4 T cell activation; binding of TCR to specific MHC-I or MHC-II receptors respectively and the presence of CD80/86 To state the cytokines for the polarization of Th cells to Th1, Th2, or regulatory T cells
8	To describe the signals for B cell activation (MHC-II binding to TCR; CD40L binding to CD40) and the process of affinity maturation To state the cytokine (IFN-γ) for the class-switching to produce IgG

### Analysis of pretest and posttest responses

Identical pretests and posttests comprised five short-answer questions, as shown in Table III. Questions 1, 3, and 4 had two subquestions each and were categorized as nonprocedural, as they assessed students on factual and conceptual knowledge. Questions 2 and 5 were categorized as procedural, as they focused on procedural knowledge, that is, steps in a process. Twenty-two and 25 students completed the pretest and posttest from the experimental and control group, respectively. Responses were marked by a single assessor according to a marking scheme.

**Table III.** Questions to assess content knowledge

No.	Question	Marking Scheme	Types of Knowledge
1	State the cellular location where peptides are loaded onto MHC complex in the following pathways: a) Endocytic pathway (via MHC-II) b) Cross-presentation (via MHC-I)	a) Phagolysosome (one mark) b) Endoplasmic reticulum (one marks)	Factual
2	Arrange the following statement in the order of when they take place during B cell activation: a) B cell undergoes affinity maturation b) B cell differentiates into Ab-producing plasma cells c) BCR presents viral Ag to T cells, leading to the activation of B cells d) BCR binds to foreign Ag, resulting in the endocytosis of foreign Ags	d, c, b, a (two marks)	Procedural
3	Describe one function of the following cytokine: a) TGF-β b) IFN-γ	a) Differentiation of CD4 T cells to regulatory T cells or inhibition of effector T cells (one mark) b) Inhibits viral replication or inhibits differentiation of Th2 cells or activation of macrophages or increase Ag presentation via MHC-I (one mark)	Factual
4	Discuss two likely consequences if an individual has a defect in CD40L	Defect in B cell activation or defect in macrophage activation or defect in class-switching (any two; one mark each)	Conceptual
5	Describe the processes involved in the activation of naive CD4 T cells to effector Th1 cell	Binding of pMHC-II on Ag presenting cells to the TCR (one mark) Binding of CD80/86 to CD28 (one mark) Activation and proliferation of CD4 T cells (1 mark) Activated CD 4 T cells will differentiate into Th1 cell in the presence of IL-12 (one mark)	Procedural

Five questions were used in the pretest and posttest to assess content knowledge.

The data were analyzed for normality. As some of the data did not have a normal distribution (there were a number of students scoring 0 for pretest questions), nonparametric tests were used. Students’ individual pretest and posttest scores were compared for both groups using a Wilcoxon signed-rank test. The experimental and control groups’ scores were analyzed using a Quade rank analysis of covariance, with the pretest scores as the covariate. Owing to the small sample size, the effect size calculations were also included in the results. The calculation used for effect size was *Z*/(square root of 2*n*), where *n* represents the number of cases (33).

### Articulation of scientific terms

Video recordings of the discussions for the five experimental and six control groups were captured and transcribed. The pattern of discussions between the gameplay and discussion segments were analyzed, and the instances of articulation of scientific terms were recorded. Scientific terms were tabulated into a master list to standardize the list of accepted terms. The master list consisted of 62 unique scientific terms (Supplemental Table II). The 11 transcripts were then cross-checked with the master list to ensure that the scientific terms were consistently scored. Total and unique counts of scientific terms were determined.

### Correlation of scientific articulation with learning gains

Group scores for the pretests and posttests were calculated by adding the individual scores of all three members. The group score for learning gains was calculated by subtracting the pretest group score from the posttest group score. Spearman’s rho correlation coefficients (ρ) were calculated to assess the level of correlation between the total number of scientific terms, unique scientific terms, and learning gains (group score). Power analysis was performed for Spearman correlation using IBM SPSS (Statistical Package for the Social Sciences).

## Results

### Collaborative digital game-based learning enhances procedural knowledge

Pretests and posttests were conducted to establish whether digital game-based learning led to better learning gains compared with viewing a video when conducted in an online collaborative setting. The mean total posttest scores were significantly higher than the mean total pretest scores for both the experimental and control groups (*p* < 0.001), indicating that both playing a game and watching a video in a virtual classroom enhanced learning (Table IV). Questions were then further categorized into nonprocedural or procedural. The posttest scores for both the nonprocedural and procedural categories were significantly higher than the pretest scores in both the experimental and control groups. When the posttest scores were compared between the experimental and control groups with pretest scores as a covariate (Table V), the posttest scores of questions on procedural knowledge were significantly higher in the experimental group (*p* = 0.006), indicating that playing the game improved procedural knowledge more than viewing the video. There were no significant differences between the experimental and control groups for the total as well as nonprocedural posttest scores.

**Table IV.** Results of Wilcoxon signed-rank test showing the difference between the pretest and posttest of knowledge assessments for both treatments

		Pretest		Posttest		*Z* (Posttest − Pretest)	*p* Value	Effect Size, *r* Value
Groups	*n*	Mean	SD	Mean	SD			
Experimental								
Total	22	3.82	2.22	7.23	2.20	−3.56	<0.001	0.537
Nonprocedural	22	2.82	1.79	3.68	1.25	−3.48	0.034	0.525
Procedural	22	1.00	1.35	3.55	1.22	−2.89	<0.001	0.436
Control								
Total	25	4.52	2.35	6.76	1.86	−4.06	<0.001	0.574
Nonprocedural	25	2.92	1.71	4.12	1.62	−2.12	0.001	0.300
Procedural	25	1.60	1.38	2.64	1.25	−3.74	0.004	0.529

**Table V.** Quade rank analysis of covariance by experimental versus control group with pretest scores as covariate

Dependent Variable	Groups	*n*	*F*	DFH	DFE	*p* Value
Total (posttest)	Experimental	22	2.852	1	45	0.098
	Control	25				
Nonprocedural (posttest)	Experimental	22	2.148	1	45	0.150
	Control	25				
Procedural (posttest)	Experimental	22	8.192	1	45	0.006
	Control	25				

DFE, degrees of freedom for error; DFH, degrees of freedom for the hypothesis.

### Scientific articulation is correlated with enhanced learning during game-based learning

To investigate the effects of collaborative game-based learning on learning outcomes, video recordings of the group discussions for the experimental and control groups were analyzed. The total and unique counts of scientific terms articulated by each of the groups, as well as the learning gains categorized by scores for total, nonprocedural, and procedural questions, are shown in Table VI. Total counts of scientific terms articulated by the experimental groups were positively correlated with total learning gains (ρ = 1.000, *p* < 0.05, power = 1) (Table VII). There was no correlation between scientific articulation and learning gains for the control group. As total counts of scientific terms had a stronger correlation with learning gains in experimental groups, it was used in the subsequent analysis.

**Table VI.** Group scores for learning gains, total counts, and unique counts of scientific terms

Groups	Learning Gains (Total)	Learning Gains (Nonprocedural)	Learning Gains (Procedural)	Scientific Articulation (Total Count)	Scientific Articulation (Unique Counts)
Experimental					
Game 1	13	6	7	342	40
Game 2	9	0	9	138	38
Game 3	12	−1	13	249	54
Game 4	5	−1	6	135	32
Game 5	3	2	1	78	25
Control					
Video 1	1	1	0	137	43
Video 2	7	2	5	144	37
Video 3	5	1	4	135	36
Video 4	10	6	4	192	39
Video 5	15	10	5	111	39
Video 6	7	4	3	15	14

**Table VII.** Spearman rho correlation coefficient between learning gains and scientific articulation

	Learning Gains		
Groups	Total	Nonprocedural	Procedural
Experimental			
Scientific terms (total)	1.000**^,a^	0.205	0.700
Scientific terms (unique)	0.900*	−0.154	0.900*
Control			
Scientific terms (total)	−0.029	−0.116	0.147
Scientific terms (unique)	0.000	0.059	−0.090

* *p* < 0.05,

** *p* < 0.01.

a Power = 1.

### Observations of scientific articulation for experimental groups

**FIGURE 3.**
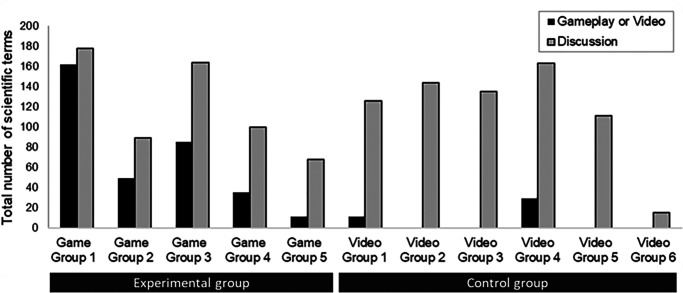
Total number of scientific terms used by the experimental and control groups during gameplay/video and discussion.

Recordings were analyzed to study whether there were differences in the group interactions for experimental and control conditions that could have led to the differences in the articulation of scientific terms and its effect on learning gains. The use of scientific terms during the game-based learning and video activity are shown in Fig. 3.

For the experimental groups, the total number of terms used was higher for the discussion segment than during the gameplay segment for all groups. The analysis revealed three main patterns of scientific articulation for the experimental condition involving a 40-min gameplay session and a 20-min postgame reflective group discussion. Excerpts from game groups 1, 4, and 5 were selected to illustrate the different patterns of scientific articulation. Game groups 2 and 3 showed intermediate characteristics between patterns 1 and 2 described below.

### Pattern G1: usage of scientific terms during gameplay and group discussion

**FIGURE 4.**
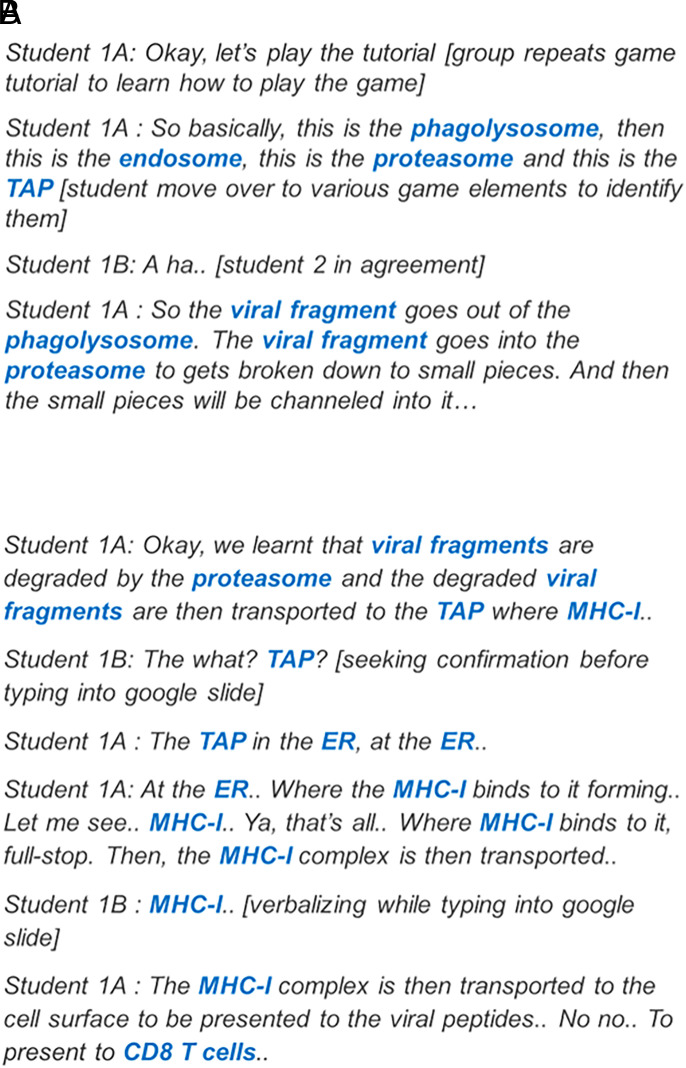
Excerpts from transcript of game group 1. Excerpts were obtained from transcript of game group 1 while the group was playing challenge 6 (**A**) or during the group discussion on challenge 6 (**B**).

Game group 1 used scientific terms during both the gameplay and group discussion segments. The group also had the highest number of scientific terms and learning gains (Table VI). Game group 1 actively tried to understand how the game worked by repeating the game tutorial and relating each game element to the actual immune component highlighted in the tutorial. Consequently, game group 1 had the highest count of scientific terms during the gameplay session (Fig. 4A). The group discussion segment went smoothly as they were able to complete most of the tasks without going back to the game (Fig. 4B). Excerpts from game group 1’s transcripts are shown below. Formal scientific terms are represented in blue whereas casual, informal terms are in red.

### Pattern G2: limited use of scientific terms during gameplay while group discussion stimulated increased articulation of scientific terms

**FIGURE 5.**
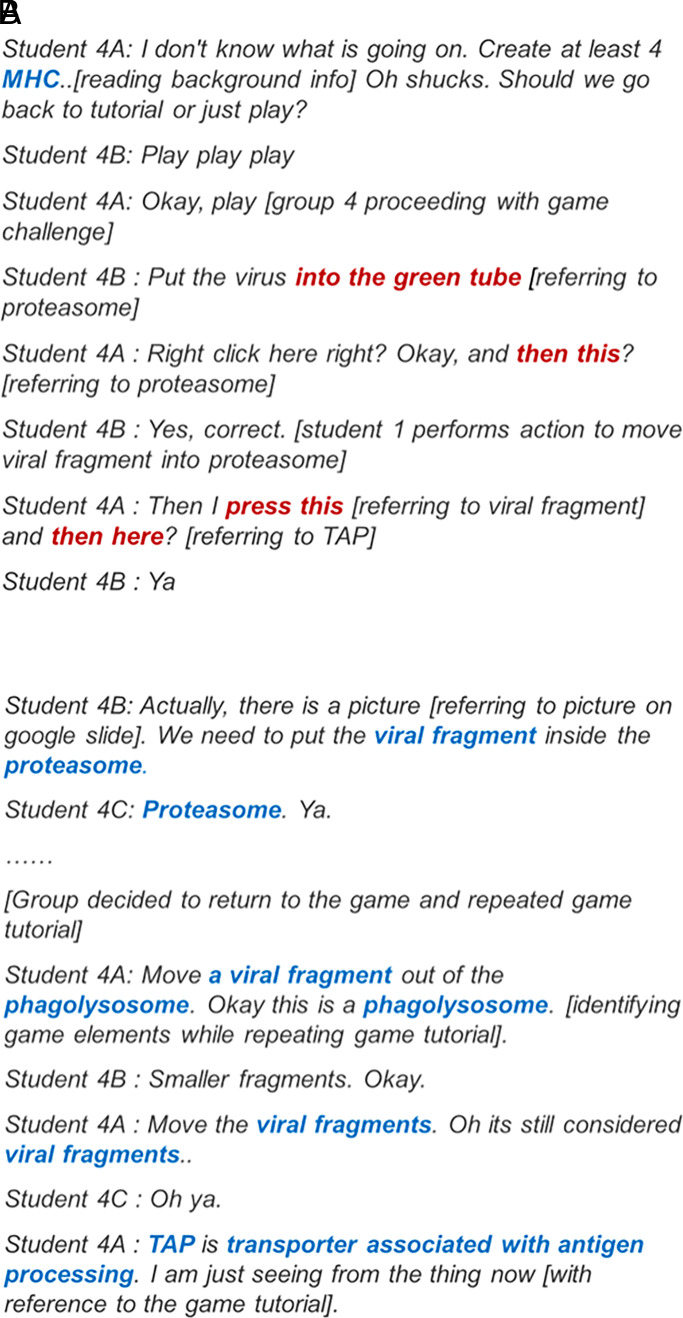
Excerpts from transcript of game group 4. Excerpts were obtained from transcript of game group 4 while the group was playing challenge 6 (**A**) or during the group discussion on challenge 6 (**B**).

Game group 4 had a low number of scientific terms articulated during the gameplay segment but higher articulation of terms during the group discussion. Analysis of the transcript showed that while game group 4 had some formal articulation of scientific terms during the gameplay session, group members had the tendency to describe the game elements based on their appearance in the game (e.g., green tube) and through the use of pronouns (e.g., this) or adverbs (e.g., here) (Fig. 5A). Despite the lack of scientific articulation, game group 4 was motivated to complete game challenges and attempted to memorize game movements to pass the game challenges. When game group 4 progressed to the group discussion, they initially had difficulty stating what was learned from the game. The group managed to complete the activity by repeating the game tutorial. The excerpts from gameplay and group discussion segments of game group 4 illustrated the higher usage of formal scientific terms during the group discussion compared with during gameplay (Fig. 5A, 5B).

### Pattern G3: limited use of scientific terms during gameplay and group discussion

**FIGURE 6.**
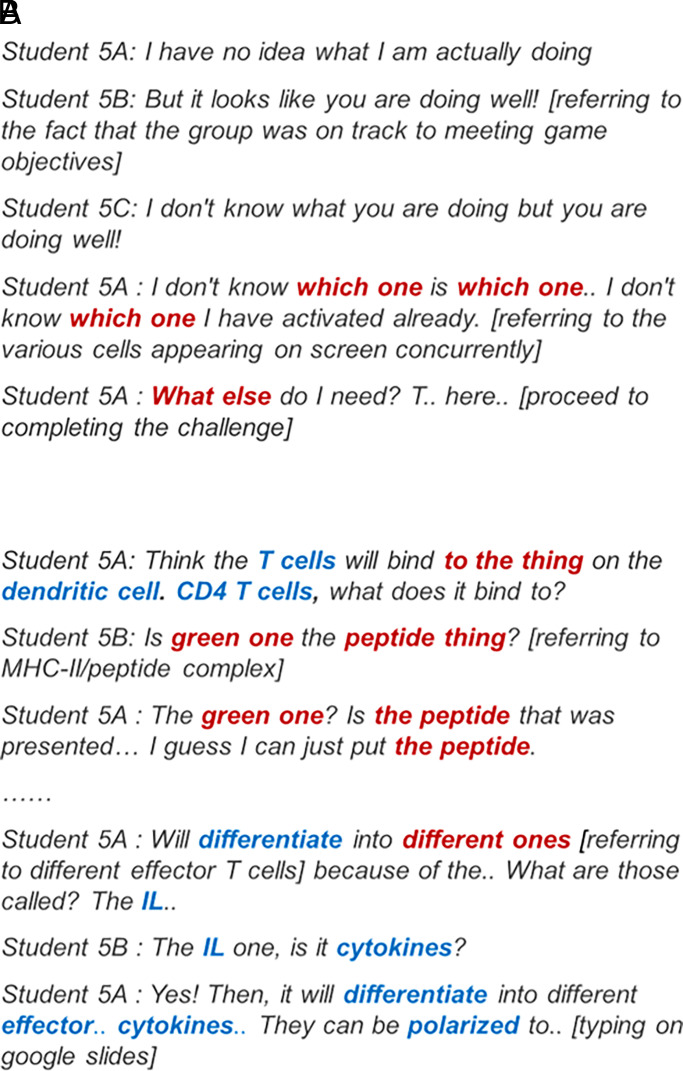
Excerpts from transcript of game group 5. Excerpts were obtained from transcript of game group 5 while the group was playing challenge 7 (**A**) and during the group discussion on challenge 7 (**B**).

Game group 5 had the lowest number of scientific terms articulated during both the gameplay (Fig. 6A) and group discussion segment (Fig. 6B). It was also noted that game group 5 had the lowest learning gains among the five groups (Table VI). Game group 5 had limited use of scientific terms when playing the game, and the conversation between group members showed that they had little understanding of the processes shown in the challenges. Similar to game group 4, game group 5 had the tendency to use descriptive words (e.g., the green one) in place of actual scientific terms. Despite this, game group 5 had one of the highest scores for the game challenges. During the group discussion, there was some articulation of scientific terms, albeit limited. In addition, the terms used were broad in nature (e.g., stating the broad category of cytokines but did not list down the different cytokines that were involved), as the group did not think it was necessary to go into specifics.

### Observations of scientific articulation for control groups

**FIGURE 7.**
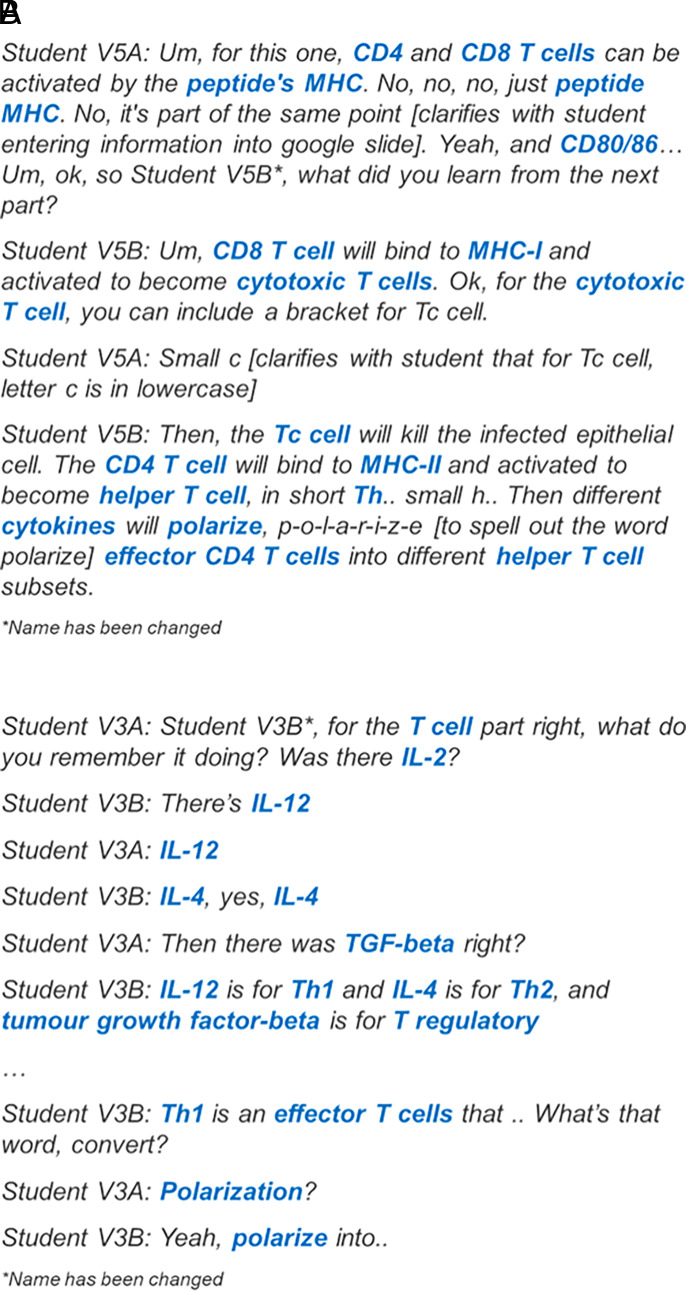
Excerpts from transcript of video group 5 and video group 3. Excerpts were obtained from transcript of video group 5 showing two students dictating information during the discussion segment for challenge 7 (**A**) and video group 3 demonstrating collaborative sense making during the discussion segment for challenge 7 (**B**).

There was little or no discussion while students were viewing the videos. Discussions took place during the 20-min postvideo documentation segment. Unlike the experimental groups with three patterns of scientific articulation, there was only one pattern of scientific articulation for the control groups. With the exception of video group 6, video groups 1–5 used formal scientific terms most of the time and rarely described the immune components based on their appearance in the video. In video groups 1–4, although there were instances of scientific collaboration, the group discussion segment was dominated by one student dictating the information that he or she recalled from the video while another student entered the information into the Google slide. For video group 5, there were two students providing information to the group, and for video 6, there was minimal discussion as students worked on the activity individually. The excerpt from the video group 5 shows students dictating the information required to complete the assigned task whereas the excerpt from video group 3 shows an example of collaboration between students (Fig. 7A, 7B).

## Discussion

*Mission: Immunity!* was previously shown to enhance the learning outcomes of students when implemented in a physical classroom setting (14). Although students played the game individually, there was discussion between students as they sought help from their peers and shared tips on how to pass the challenges. However, peer interactions were mainly focused on the gameplay and seldom contributed to the intended learning outcomes. Thus, we saw a need to intentionally design a learning activity around the single-player game, for students to engage in discussion on the learning content. In this study, the learning activity was redesigned to incorporate collaborative discussion after the gameplay segment, where students were to record their key learning points from the game in a collaborative document. As this study was implemented during the COVID-19 pandemic when online learning was mandated, collaborative learning also brought in the social presence to an otherwise isolated online gameplay experience. Findings from this study showed that *Mission: Immunity!* was effective in enhancing learning outcomes when it was implemented in a virtual classroom and in a collaborative manner.

### Collaborative sense-making is crucial for game-based learning but less critical for video activity

As student collaboration provides opportunities for students to be engaged in verbal reasoning, it may enable students to undergo a process of cognitive restructuring that brings about conceptual learning (34). Hence, recordings of the game-based learning and video activities were analyzed on the extent of scientific articulation as well as the nature of verbal collaborations of participants.

The positive correlation between the extent of scientific articulation and learning gains during collaborative game-based learning suggests the importance of collaborative sense-making in game-based learning. Analysis of the scientific terms used revealed a difference in the extent of scientific articulation between gameplay and postgame discussion. With the exception of game group 1, groups generally demonstrated a lower extent of scientific articulation during gameplay compared with the postgame discussion. This could be attributed to high cognitive load of the learning content as well as the design of game challenges in *Mission: Immunity!* (35, 36). As students were focused on playing the game, there could have been less cognitive resources available to acquire knowledge and partake in discussion. However, this also indicates that playing games as a standalone activity may not result in content learning. Conversations and observations of game groups 4 and 5 during gameplay sessions exemplified this, as groups were able to complete the game despite having little understanding of the subject matter. As students have the tendency to use trial and error (37) and heuristics to achieve game objectives (38), emphasis should be placed on activity design so that students could gain domain-specific knowledge and skills from playing the game (39, 40). To address this, the learning activity was designed to include a postgame group discussion where students were tasked to discuss what they had learned and write a summary of the key points. During this activity, there was a higher level of articulation of scientific terms, suggesting the ability of the postgame group discussion to promote acquisition of scientific concepts. On a similar note, Garris et al. (18) emphasized the need for a postgame debrief to help learners bridge the gameplay experience to real world learning.

Unexpectedly, there was no correlation between the extent of scientific articulation and learning gains for the groups assigned to the video activity. This suggests that collaborative activities may not be required for knowledge acquisition through the viewing of a video. One possibility could be attributed to the nature of video viewing, which requires significantly less cognitive resources as compared with playing an educational game that is intrinsically complex. As high cognitive load hampers learning and transfer, knowledge acquisition from the viewing of the video may be more effective as a standalone activity as compared with the game (36). Without the need to achieve the game objectives, students could focus on the learning content in the video.

This is also supported by our observations in two distinct ways. First, all video groups tended to use formal scientific terminologies during their discussion as opposed to the game groups where most groups (game groups 2–5) referred to the immune system components by their appearance during their gameplay and discussion. In addition, it was observed that video groups faced fewer difficulties in completing the task of documenting the key learning points as one or more group members were observed to be dictating the required information most of the time. These findings suggest that knowledge acquisition had taken place primarily through viewing the video as opposed to the discussion segment. On the contrary, with exception of game group 1, most game groups were engaged in sense-making during the discussion segment to elicit the required information for documentation.

### Enhancement of procedural knowledge during game-based learning

Although both collaborative game-based learning and the viewing of prerecorded game videos brought about gains in nonprocedural and procedural knowledge, it was observed that collaborative game-based learning led to a significantly higher acquisition of procedural knowledge as compared with the viewing of a prerecorded video of the game. This likely stems from the active nature of game-based learning where learners learn by doing and are engaged in cycles of action and reflection (35, 41). By generating and experimenting with a possible game solution, as well as observing its outcome to a game problem, learners construct a schema that allows the discovery of better solutions. This in turn leads to knowledge acquisition and deeper understanding. The gains in procedural knowledge could also be attributed to the game design of *Mission: Immunity!*, as each game challenge surrounds the completion of a specific process during the course of an immune response. Unlike the control group where groups watched a series of videos, the experimental groups strove to pass the game challenges by optimizing strategies to fulfill the game objectives. Groups were also motivated by the various game elements to repeat the challenges to pass or attain a better score.

The study showed that scientific articulation during collaborative game-based learning enhanced immunology learning, particularly, in this case, for procedural knowledge, as students were required to manipulate immune system components. In addition, we have also demonstrated the importance of providing opportunities for collaborative sense-making through a postgame discussion to reinforce learning through games.

### Limitations of this study

One key limitation of the study was the small sample size, as informed consent was only obtained from a subset of the students. Another limitation is that only short-term gains were measured; the study did not examine long-term retention of learning, or subsequent access of the game by students in their own time. Additionally, as playing the game required more time than watching a gameplay video, the groups that played the game had an additional 20 min to engage with the content, which may also have led to better learning.

### Recommendations for using digital game-based learning to enhance the learning of immunology in an online environment

Although much of learning has returned to a face-to-face setting, there are settings for online learning such as in distance education. In this study, we successfully implemented game-based learning in an online collaborative setting, but some challenges were encountered. A key difficulty was the provision of timely assistance when students faced gameplay issues. Lecturers did not have visibility of all students at the same time as groups were assigned to breakout rooms. Hence, when implementing online synchronous game-based learning, one consideration would be to consider a lower student-to-instructor ratio as well as to provide detailed written instructions on the learning activity and ways to seek assistance when required. Additionally, lecturers can encourage students to keep their cameras on during the online session so that students remained engaged throughout the session.

### Recommendations for using digital game-based learning to enhance the learning of immunology in a physical classroom environment

Although it is undeniable that the implementation of digital game-based learning requires more prelesson planning and curriculum time as compared with viewing a video, it can augment learning in several ways. Digital game-based learning is an active learning approach that has been shown to improve learning of immunology (3). The findings of this study further indicate that game-based learning can improve understanding of procedural knowledge compared with just watching a gameplay video. Besides effects on learning outcomes, our earlier study demonstrated the ability of digital game-based learning to enhance student motivation and engagement (14).

For a successful implementation of game-based learning, facilitation of the learning experience is of utmost importance. Learning can be enhanced by incorporating collaborative learning activities. In addition to peer discussion and learning around the game, a postgame discussion can be included to encourage scientific articulation, which helps students to acquire the challenging terminology and promote conceptual understanding as students draw relationships between the scientific terminologies. Although the context in this study was for learning in the online environment, the findings can also be applied to learning in a traditional face-to-face classroom environment, where students are equipped with laptops.

The effectiveness of digital game-based learning is also dependent on the design of the game. In *Mission: Immunity!*, the challenge was to perform certain actions in a limited time, which lent excitement gamewise to the detriment of learning the terminologies and processes. In the current game, it is possible to play the game without necessarily learning the content or the terminology, which may be more incidental. This was also a finding for another game, *Immune Attack*, when evaluated by a group of teacher education students (42). They found that the focus was more on controlling a nanobot through the body, and they could play the game without learning the disciplinary content. However, in another study, students were able to learn concepts in *Immune Attack* just by playing the game without being explicitly taught (11). For games to improve conceptual learning, perhaps the approach could be more conceptual, with students having to make decisions, for example, on which cytokine to use for T cell polarization, and then see the consequence. That would focus more attention on the immune system components.

Creating an educational game that is subject-specific is costly and time-consuming and requires expertise in the disciplinary content, pedagogy, game design and programming. While the game *Mission: Immunity!* used in this study was proprietary, there are also immunology games that are free to play on the Internet. These games, some of which are listed in Table I, are single-player games, similar to *Mission: Immunity!*. Recommendations for improving learner outcomes using digital game-based learning include designing a gameplay experience as a collaborative group activity, and incorporating a postgame group discussion for students to articulate the concepts, procedures, and terminology, thus making the learning more explicit, and providing students with the opportunity to make use of scientific terms in the context of the postgame activity.

## Supplementary Material

Supplemental Tables 1 (PDF)

## References

[1] Faggioni, T., N. C. da Silva Ferreira, R. M. Lopes, A. A. Fidalgo-Neto, V. Cotta-de-Almeida, L. A. Alves. 2019. Open educational resources in immunology education. Adv. Physiol. Educ. 43: 103–109. 30835146 10.1152/advan.00116.2018

[2] Stranford, S. A., J. A. Owen, F. Mercer, R. R. Pollock. 2020. Active learning and technology approaches for teaching immunology to undergraduate students. Front. Public Health 8: 114. 32478022 10.3389/fpubh.2020.00114 PMC7232573

[3] Bohlson, S. S., J. J. Baty, M. C. Greenlee-Wacker, H. A. Bruns. 2022. Piecing complement together with LEGO bricks: impacts on interest, confidence, and learning in the immunology classroom. Immunohorizons 6: 488–496. 35868839 10.4049/immunohorizons.2200040

[4] Prensky, M. 2001. Digital Game-Based Learning. McGraw-Hill, New York.

[5] Gee, J. P. 2003. What Video Games Have to Teach Us About Learning and Literacy. Palgrave Macmillan, New York.

[6] Cheng, M.-T., J.-H. Chen, S.-J. Chu, S.-Y. Chen. 2015. The use of serious games in science education: a review of selected empirical research from 2002 to 2013. J. Comput. Educ. 2: 353–375.

[7] Tsai, Y.-L., C.-C. Tsai. 2020. A meta-analysis of research on digital game-based science learning. J. Comput. Assist. Learn. 36: 280–294.

[8] Brown, C. L., M. A. Comunale, B. Wigdahl, S. Urdaneta-Hartmann. 2018. Current climate for digital game-based learning of science in further and higher education. FEMS Microbiol. Lett. 365: 1–10. 10.1093/femsle/fny237 PMC6203454 30260380

[9] Li, M.-C., C.-C. Tsai. 2013. Game-based learning in science education: a review of relevant research. J. Sci. Educ. Technol. 22: 877–898.

[10] Cheng, M.-T., T. Su, W.-Y. Huang, J.-H. Chen. 2014. An educational game for learning of human immunology: what do students learn and how do they perceive? Br. J. Educ. Technol. 45: 820–833.

[11] Stegman, M. 2014. Immune attack players perform better on a test of cellular immunology and self confidence than their classmates who play a control video game. Faraday Discuss. 169: 403–423. 25340640 10.1039/c4fd00014e PMC4489431

[12] Raimondi, S. L. 2016. ImmuneQuest: assessment of a video game as a supplement to an undergraduate immunology course. J. Microbiol. Biol. Educ. 17: 237–245. 27158304 10.1128/jmbe.v17i2.1060 PMC4858359

[13] Nankervis, S., G. Meredith, P. Vamplew, N. Fotimatos. 2012. Taming the devil: a game-based learning approach to teach immunology. Proceedings of ASCILITE (Australian Society for Computers in Learning in Tertiary Education) Annual Conference 2012. M. Brown, M. Hartnett, T. Stewart, eds. Wellington, NSW, Australia, p. 703–707. Available at: https://www.ascilite.org/conferences/Wellington12/2012/images/custom/nankervis%2C_scott_-_taming.pdf.

[14] Low, P. Y., G. B. Lim. 2017. Game-based learning to improve student learning and engagement in immunology. In Transactions of ISATE 2017, Singapore, Singampore. p. 683–688. Available at: http://static1.1.sqspcdn.com/static/f/1751776/27865310/1521967778820/Game+based+learning+to+improve+student+learning+and+engagement+in+Immunology.pdf?token=s2%2B2avgVvhk57NsUnpUiaR7lAJI%3D.

[15] Teng, T.-Y., W.-C. Chou, M.-T. Cheng. 2021. Learning immunology in a game: learning outcomes, the use of player characters, immersion experiences, and visual attention distribution. J. Comput. Assist. Learn. 37: 475–486.

[16] Barab, S., S. Zuiker, S. Warren, D. Hickey, A. Ingram-Goble, E. Kwon, I. Kouper, S. C. Herring. 2007. Situationally embodied curriculum: relating formalisms and contexts. Sci. Educ. 91: 750–782.

[17] Clark, D. B., B. C. Nelson, H. Y. Chang, M. Martinez Garza, K. Slack, C. M. D’Angelo. 2011. Exploring Newtonian mechanics in a conceptually-integrated digital game: comparison of learning and affective outcomes for students in Taiwan and the United States. Comput. Educ. 57: 2178–2195.

[18] Garris, R., R. Ahlers, J. E. Sriskell. 2002. Games, motivation and learning: a research and practice model. Simul. Gaming 33: 441–467.

[19] Barzilai, S., I. Blau. 2014. Scaffolding game-based learning: impact on learning achievements, perceived learning, and game experiences. Comput. Educ. 70: 65–79.

[20] Ke, F. 2016. Designing and integrating purposeful learning in game play: a systematic review. Educ. Technol. Res. Dev. 64: 219–244.

[21] Wouters, P., H. van Oostendorp. 2013. A meta-analytic review of the role of instructional support in game-based learning. Comput. Educ. 60: 412–425.

[22] Sitzmann, T. 2011. A meta-analytic examination of the instructional effectiveness of computer-based simulation games. Person. Psychol. 64: 489–528.

[23] Barab, S. A., B. Scott, S. Siyahhan, B. Goldstone, A. Ingram-Goble, S. J. Zuiker, S. Warren. 2009. Transformational play as a curricular scaffold: using videogames to support science education. J. Sci. Educ. Technol. 18: 305–320.

[24] Chen, C., K. Wang, Y. Lin. 2015. The comparison of solitary and collaborative modes of game-based learning on students’ science learning and motivation. J. Educ. Technol. Soc. 18: 237–248.

[25] Sung, H.-Y., G.-J. Hwang. 2013. A collaborative game-based learning approach to improving students’ learning performance in science courses. Comput. Educ. 63: 43–51.

[26] Eaton, G. V., B. D. Clark, B. E. Smith. 2015. Patterns of physics reasoning in face-to-face and online forum collaboration around a digital game. Int. J. Educ. Math. Sci. Technol. 3: 1–13.

[27] Srisawasdi, N., P. Panjaburee. 2019. Implementation of game-transformed inquiry-based learning to promote the understanding of and motivation to learn chemistry. J. Sci. Educ. Technol. 28: 152–164.

[28] Annetta, L. A., M. Cheng, S. Holmes. 2010. Assessing twenty-first century skills through a teacher-created video game for high school biology students. Res. Sci. Technol. Educ. 28: 101–114.

[29] Schwartz, D. 1995. The emergence of abstract representations in dyad problem solving. J. Learn. Sci. 4: 321–354.

[30] Yun, E. 2020. Correlation between concept comprehension and mental semantic networks for scientific terms. Res. Sci. Technol. Educ. 38: 329–354.

[31] Krontiris-Litowitz, J. 2009. Articulating scientific reasoning improves student learning in an undergraduate anatomy and physiology course. CBE Life Sci. Educ. 8: 309–315. 19952099 10.1187/cbe.08-11-0066 PMC2786281

[32] Low, P. Y., C. M. Teoh, G. B. Lim. 2019. Augmenting learning of immunology through an online learning package and a digital game—what’s next? In Proceedings of ASCILITE (Australian Society for Computers in Learning in Tertiary Education) Annual Conference 2019. Y. W. Chew, K. M. Chan, A. Alphonso, eds. Singapore, Singapore, p. 503–508. Available at: https://2019conference.ascilite.org/assets/papers/Paper-071.pdf.

[33] Pallant, J. 2020. SPSS Survival Manual. Routledge, London.

[34] Kagan, S. 2014. Kagan structures, processing, and excellence in college. J. Excell. Coll. Teach. 29: 119–138.

[35] Kiili, K. 2005. Digital game-based learning: towards an experiential gaming model. Internet High. Educ. 8: 13–24.

[36] Sweller, J., J. J. G. van Merrienboer, F. Paas. 2019. Cognitive architecture and instructional design: 20 years later. Educ. Psychol. Rev. 31: 261–292.

[37] Chang, H.-Y., C. Quintana, J. Krajcik. 2010. The impact of designing and evaluating molecular animations on how well middle school students understand the particulate nature of matter. Sci. Educ. 94: 73–94.

[38] Turkle, S. 1997. Seeing through computers: education in a culture of simulation. Am. Prospect 31: 76–82.

[39] Ke, F. 2009. A qualitative meta-analysis of computer games as learning tools. In Handbook of Research on Effective Electronic Gaming in Education, Vol. 1. R. Ferdig, ed. IGI Global, Hershey, PA, p. 1–32.

[40] Leutner, D. 1993. Guided discovery learning with computer-based simulation games: effects of adaptive and non-adaptive instructional support. Learn. Instr. 3: 113–132.

[41] Squire, K. 2006. From content to context: videogames as designed experience. Educ. Res. 35: 19–29.

[42] Barendregt, W., M. von Feilitzen. 2010. Attacking Immune Attack? An evaluation by teacher students. In Proc. 4th European Conference on Games-Based Learning. Copenhagen, Denmark.

