# Supplementary material for: Scientific Articulation during Collaborative Digital Game-Based Learning Enhances Learning of Immunology
Source: Immunohorizons. 2023 Nov 1;7(11):718–28. doi: 10.4049/immunohorizons.2300004 (PMC10695416; doi:10.4049/immunohorizons.2300004)
Supplement: Supplemental Tables 1 (PDF) [file IH_2300004_Supplemental_1.pdf]

Supplementary Table 1 – Pre-recorded gameplay videos for Challenges 6 - 8

VIDEO	VIDEO LINK
**CHALLENGE 6**	https://drive.google.com/file/d/1R-OnhQ2K_sR5OrTckpIpU8I-aDellcpp/view?usp=sharing
**CHALLENGE 7**	https://drive.google.com/file/d/1F6FYh_O-j6f0EJhYl4Yrf3vPV16EPWAs/view?usp=sharing
**CHALLENGE 8**	https://drive.google.com/file/d/1us3y-INmyTHrBBc4Q-OuHwflv3FJCuSD/view?usp=sharing

Supplementary Table 2 – List of accepted scientific terminologies

SCIENTIFIC TERMINOLOGIES	ACCEPTED VARIATION
**AFFINITY**	affinity
**AFFINITY MATURATION**	affinity maturation
**ANTIBODIES**	antibodies, antibody
**ANTIGEN**	antigen
**ANTIGEN PRESENTING PATHWAYS**	antigen presenting pathways, antigen presenting
**B CELL**	B cell, B cells
**BCR**	BCR
**CD28**	CD28
**CD4 T CELL**	CD4 T cell, CD4, CD4 T cells
**CD40**	CD40
**CD40L**	CD40L
**CD8 CELL**	CD8 T cell, CD8, CD8 cell, CD8 T cells
**CD80/86**	CD80/86, CD80, CD86
**CLASS-SWITCHING**	class-switch, class-switching
**CROSS-PRESENTATION**	cross-presentation
**CYTOKINES**	cytokines
**CYTOPLASM**	cytoplasm
**CYTOTOXIC T CELL**	cytotoxic T cell, cytotoxic T cells, cytotoxic cells, Tc cells
**DENDRITIC CELL**	dendritic cell, dendritic cells
**DIFFERENTIATION**	differentiation, differentiate
**EFFECTOR T CELLS**	effector T cells, effector CD4 T cells, effector cells
**ENDOCYTOSE**	endocytose
**ENDOPLASMIC RETICULUM**	endoplasmic reticulum, ER
**ENDOSOME**	endosome
**EPITHELIAL CELL WALL**	epithelial cell
**FOLLICULAR DENDRITIC CELL**	follicular dendritic cell
**GERMINAL CENTRE**	germinal centre
**IFN-GAMMA**	IFN-gamma, IFN, IFN-g
**IG**	Ig
**IGA**	IgA
**IGG**	IgG
**IGM**	IgM
**INTERLEUKIN**	IL, interleukin
**IL-12**	IL-12
**IL-2**	IL-2
**IL-4**	IL-4
**LYSOSOME**	lysosome
**M CELLS**	M cells
**MHC**	MHC, MHC receptor, peptide:MHC, pMHC
**MHC-I**	MHC-I, MHC-I complexes, pMHC-I
**MHC-II**	MHC-II, p-MHC-II
**PHAGOCYTOSE**	phagocytose
**PHAGOLYSOSOME**	phagolysosome, phagolysosomes
**PHAGOSOMES**	phagosomes
**PLASMA CELL**	plasma cell, plasma cells, plasma (in context of game)
**POLARISATION**	polarised, polarise, polarisation
**PRIMARY FOLLICLE**	primary follicle, follicle
**PROTEASOME**	proteasome
**T CELL**	T cell, T cells
**T REGULATORY CELL**	T regulatory, T regulatory cell, T regulatory cells, T-reg, T regs, T-reg cell
**TAP**	TAP, transporter associated with antigen processing
**TCR**	TCR
**TGF-B**	TFG-b, TGF-beta, TGF
**TH CELL**	TH cell
**TH1 CELL**	TH1 cell, TH1 cells, TH1
**TH2**	TH2
**VESICLES**	vesicles
**VIRAL FRAGMENT**	viral fragment, viral fragments
**VIRAL PEPTIDE**	viral peptide, viral peptides
